# A Novel Method for Growing α-Ga_2_O_3_ Films Using Mist-CVD Face-to-face Heating Plates

**DOI:** 10.3390/nano13010072

**Published:** 2022-12-23

**Authors:** Yan Zuo, Qian Feng, Tao Zhang, Xusheng Tian, Wenji Li, Jiale Li, Chunfu Zhang, Jincheng Zhang, Yue Hao

**Affiliations:** 1 State Key Discipline Laboratory of Wide Bandgap Semiconductor Technology, School of Microelectronics, Xidian University, Xi’an 710071, China; 2 Shaanxi Joint Key Laboratory of Graphene, School of Microelectronics, Xidian University, Xi’an 710071, China

**Keywords:** α-Ga_2_O_3_ film, mist-CVD, X-ray diffraction, face-to-face

## Abstract

In this paper, the method for growing α-Ga_2_O_3_ films on c-plane sapphire substrates using an inexpensive fine-channel mist-CVD face-to-face heating plate was investigated. Because high temperatures can result in reactor deformation, expensive AlN ceramics resistant to deformation are used as the reactor fabrication material in traditional fine-channel mist-CVD equipment, which limits its use for promotion and research purposes. In this work, we used a face-to-face heating method to replace the traditional single-sided heating method which will reduce the requirement for equipment sealability. Therefore, cheap quartz can be used to replace expensive AlN ceramics to make reactors, which can greatly reduce the cost of mist-CVD equipment. We also investigated the effects of substrate temperature and carrier gas on the crystalline quality and surface morphology of α-Ga_2_O_3_ films. By optimizing the fabrication conditions, we obtained triangular grains with edges that were clearly visible in atomic force microscopy images. Using absorption spectrum analysis, we also found that the optical bandgap of the film reached 5.24 eV. Finally, we recorded a value of 508 arcsec for the full width at half maximum of the α-Ga_2_O_3_ (0006) diffraction peak in the X-ray diffraction pattern.

## 1. Introduction

Gallium oxide (Ga_2_O_3_) is an ultrawide bandgap (UWBG) semiconductor material which has attracted much attention in the fields of power electronics and solar-blind UV photodetectors [1,2,3,4,5,6,7,8,9,10]. Compared with other UWBG semiconductor materials such as SiC and GaN, Ga_2_O_3_ has a bandgap of over 4.9 eV and a high breakdown critical field of over 8 MV/cm [1,3,5], which make it better suited for application in UV solar-blind photodetectors [6,8,9,10], high-power devices [11,12,13,14], and transparent conductive layers [15,16]. Generally, several distinct Ga_2_O_3_ polymorphs (α, β, ε, γ, δ, and κ) can be crystallized [17,18,19,20]. Among these, β-Ga_2_O_3_ is a thermodynamically stable phase, and the others are metastable phases, which means that the others phase can easily transform into the β phase under high-temperature conditions. β-Ga_2_O_3_ readily exhibits single-crystal growth, which means that homoepitaxy can be achieved, and the incidence of structural defects is reduced. However, the lattice structure of β-Ga_2_O_3_ belongs to the C2/m space group, which is characterized by a highly asymmetric monoclinic structure [21,22]. This causes significant anisotropy in the thermal conductivity and optical properties of β-Ga_2_O_3_. High-quality β-Ga_2_O_3_ can only be obtained by homoepitaxy, which limits the application of this material. For this reason, the study of Ga_2_O_3_ has expanded to metastable phases with higher symmetry, such as corundum α-Ga_2_O_3_, with a bandgap of 5.3 eV. The crystal structure of α-Ga_2_O_3_ is an R$\bar{3}$C space group, which is the same as the crystal structure of α-Al_2_O_3_ (sapphire substrate), with lattice mismatch rates of 4.6% and 3.3% in the a- and c-axis directions, respectively [23]. Therefore, high-quality α-Ga_2_O_3_ films can be deposited upon inexpensive sapphire substrates, greatly reducing the cost of α-Ga_2_O_3_ production. The crystal structure of α-Ga_2_O_3_ is similar to that of α-M_2_O_3_ (M = Ir, In, Cr, Al, etc.), so α-Ga_2_O_3_ can form alloys with these materials [23,24]. Ga_2_O_3_ cannot be used in p-type doping, which limits its potential use in MOSFET devices; however, p-type doping of α-Ir_2_O_3_ and α-Cr_2_O_3_ has been realized, so n-type-doped α-Ga_2_O_3_ can form a heterojunction with p-type-doped α-Ir_2_O_3_ and α-Cr_2_O_3_ [25]. Finally, α-Ga_2_O_3_ films can be grown by various methods, such as metalorganic chemical vapor deposition (MOCVD) [26,27,28], hydride vapor phase epitaxy (HPVE) [28,29,30,31,32], and mist chemical vapor deposition (mist-CVD) [33,34,35]. With the development of α-Ga_2_O_3_ research, α-Ga_2_O_3_ films can also be obtained by other growth methods in the future, such as pulsed laser deposition (PLD) [36,37], electrochemical deposition [38,39], molecular beam epitaxy (MBE) [40].

Mist-CVD is a simple, economical, and safe technique for film growth. In 2008, Shinohara et al. reported a method for growing α-Ga_2_O_3_ films using a hot-wall mist-CVD method in which the reactor consisted of a common tube furnace that could be mass-produced [33]. However, it is difficult to obtain large and uniform films. In 2012, Kawaharamura et al. reported a fine-channel (FC-type) mist-CVD method for growing Sn-doped α-Ga_2_O_3_ films through a narrow reaction space to improve the film formation efficiency [34]. By this method, uniform films with a large area can be efficiently grown, resulting in lower levels of reaction source waste. However, a high-sealability environment is required for this method, and the materials used to make the reactor cannot decompose at high temperatures to generate impurities. Therefore, expensive AlN ceramics are required as the reactor fabrication material in the traditional FC-type mist-CVD method [34], which increases the production cost of the system and limits its potential dissemination for research purposes. 

In this paper, we propose a face-to-face heating technique based on the FC-type mist-CVD and describe the equipment to carry out a new method. We also investigate the effects of growth temperature and carrier gas on the crystalline quality of the fabricated films. The face-to-face heating method reduces the high-sealability requirements of the equipment during film growth, so we were able to use inexpensive high-temperature resistant quartz in place of expensive ALN ceramics. As the result, our equipment reduces the costs associated with production. We used a rear fan in place of dilution gas, greatly reducing carrier gas consumption. We also surrounded our reactor with an inexpensive water-cooled outer wall that cannot deform under high temperatures. Compared with the procedure described in [30], the method described in this paper requires equipment of considerably lower cost. By optimizing our experimental conditions, we grew α-Ga_2_O_3_ film with high crystal quality using our mist-CVD face-to-face heating equipment.

## 2. Experimental Section

**Figure 1:**
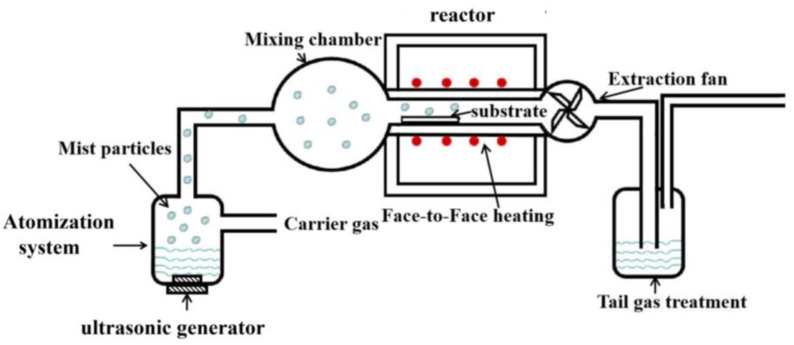
Mist-CVD face-to-face heating mist-CVD equipment system.

Figure 1 shows the mist-CVD equipment system used in our experiments, which consists of an atomization system and a modified homemade fine-channel (FC) reactor. We built a 2.4 MHz ultrasonic generator into the atomization system to generate <3 μm of mist droplets, which were mixed in the mixing chamber and transported to the reactor by the carrier gas, and the gap height of the reactor was 2 mm. During the growth process, the temperature of the upper hot plate was set at 20 °C higher than that of the lower hot plate to increase the downward velocity of the mist droplets. An adjustable power fan was installed at the end of the reactor to reduce the accumulation of mist droplets at this location. Through these methods, the effective growth space in the reactor was greatly increased. 

The α-Ga_2_O_3_ films were grown on 1 cm **×** 1 cm sapphire substrates using face-to-face heating mist-CVD equipment. Table 1 shows the experimental conditions of α-Ga_2_O_3_ films grown at different temperatures and with different carrier gases. We used 0.03 mol/L gallium acetylacetonate in deionized water as the Ga source, and we added 1% HCl to completely dissolve all gallium acetylacetonate. The sapphire substrates were immersed in a mixture of ethanol, acetone, and deionized water, which were vibrated with ultrasonic waves for 30 min and blown dry with a nitrogen gun. Air or ultrahigh-purity N_2_ was used as the carrier gas to transport the mist droplets into the reactor, and the growth time for all samples was 45 min. 

The crystal structure of α-Ga_2_O_3_ thin films was characterized using X-ray diffraction (XRD) (D8 Advance, Bruker, Karlsruhe, Germany) with Cu Kα radiation (Kα_1_ = 1.54056 Å, Kα_2_ = 1.5418 Å) for scanning θ-2θ at a scan speed of 8 (degrees/minute). The optical transmittance and absorption spectra were measured using a lambda 950 dual-beam UV–Vis spectrophotometer to analyze the optical properties and optical bandgaps. The film morphology was characterized by atomic force microscopy (AFM) (Agilent 5500, Palo Alto, CA, USA).

## 3. Results and Discussion 

**Figure 2:**
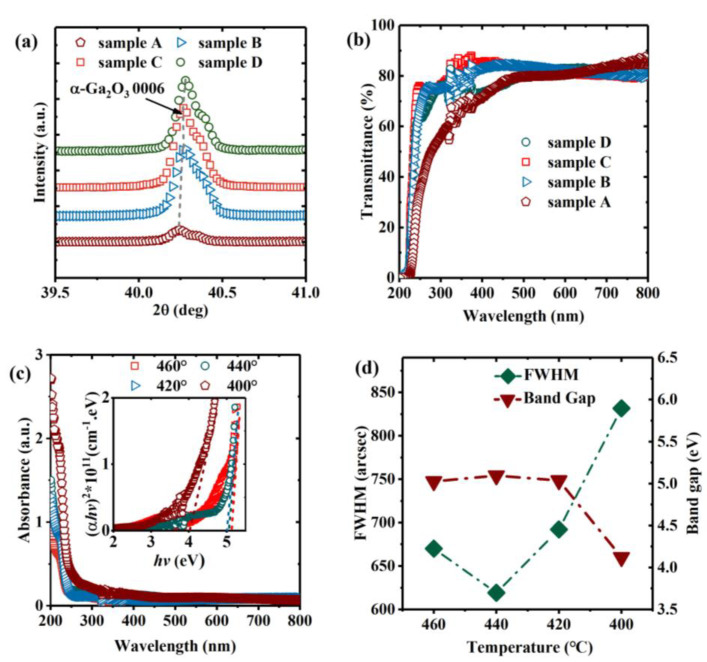
(**a**) XRD θ-2θ diffraction profiles of α-Ga_2_O_3_ films; (**b**) optical transmittance, (**c**) optical absorption spectra, (**d**) bandgaps, bandgap errors, and FWHMs of α-Ga_2_O_3_ films.

**Figure 3:**
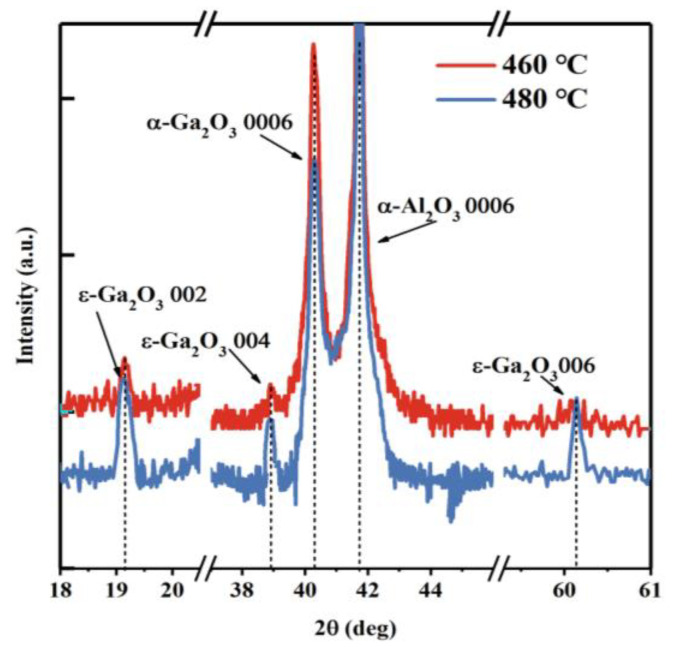
XRD θ-2θ diffraction profiles of Ga_2_O_3_ thin films grown at 460 °C and 480 °C.

Figure 2a shows XRD 2θ diffraction profiles for samples A, B, C, and D prepared at 400–460 °C. The X-ray diffraction peaks at 40.264° are for the α-Ga_2_O_3_ 0006 (PDF#06-0503). The asymmetry of diffraction peak shapes in Figure 2a is the double line broadening effect caused by Kα radiation in the XRD 2θ measurements. The α-Ga_2_O_3_ 0006 diffraction peaks can be observed in all samples, but the diffraction peak of sample A is shifted by a low angle, and sample D also shows a slight and high-angle shift. According to the Bragg diffraction formula [36]:

2*d*sin*θ* = *nλ* (1)

where *d* represents the plane spacing of the thin film atomic layer, *θ* represents the Bragg diffraction angle, and *λ* represents the X-ray wavelength of the XRD equipment, The plane strain ε_z_ in the lattice along the c-axis in α-Ga_2_O_3_ films deposited on different substrate temperatures is estimated [36,37], as follows:

(2) $$\varepsilon_{z}=\frac{c-c_{0}}{c_{0}}\times 100\%$$

where *c*_0_ is the c-axis lattice constant of α-Ga_2_O_3_ film in unstrained. We calculated that sample A exhibited 0.0727% strain, indicating that the low-temperature growth causes an increase in the c-axis lattice constant of the α-Ga_2_O_3_ film. Moreover, sample D exhibited −0.003% strain, the negative sign indicates that the c-axis lattice constant decreases due to high-temperature growth. In addition, when the temperature rises above 460 °C, we found small peaks of ε-Ga_2_O_3_ 002, 004, 006 at 19.124°, 38.943°, and 60.081° (PDF#06-0509), as shown in Figure 3. The intensity of the diffraction peak of ε-Ga_2_O_3_ increases with the increase in growth temperature, which indicates that polycrystalline Ga_2_O_3_ thin films begin to appear. Figure 2b,c shows the optical transmittance and absorbance spectra, respectively, in the wavelength range of 200–800 nm for samples A, B, C, and D. In Figure 2b, the spectrum clearly shows a band edge shift due to the change in substrate temperature, with 75% transparency in the visible range above 250 nm. In addition, the average transmittance of sample A is significantly lower than that of samples grown under other substrate temperatures. This is because gallium acetylacetonate cannot completely decompose at lower temperatures, resulting in the formation of an amorphous film. It can be observed that the absorption edge slope of sample A is much lower than that of films grown at other temperatures. Sharp ultraviolet absorption edges at approximately 230 nm can also be found, with the absorption edge being shifted to shorter wavelengths at higher growth temperatures. Figure 2c shows the optical absorption spectra of the α-Ga_2_O_3_ films grown under different temperatures. Using Formula (3) [37]:

(*αhv*)^2^ = C (*hv* − *E_g_*) (3)

where *a* and *a*_0_ are absorption coefficients, *h* is Planck’s constant, *v* is the optical frequency, and C is a constant; we can extrapolate the linear part of the (*αhυ*)^2^ − *hυ* curve to (*hυ*)^2^ = 0 to determine the optical bandgap values of the α-Ga_2_O_3_ films grown at different temperatures. These calculated optical bandgaps are represented by the red line in Figure 2d, which also shows the full width at half maximum (FWHM) of the (0006) diffraction peaks of samples A–D. 

Among our samples, sample C had the lowest FWHM value and the highest bandgap, but when the substrate temperature decreased to 400 °C, the FWHM and bandgap values of sample A exceeded those of the other three samples. This is because the substrate temperature of sample C was closer to the formation energy of α-Ga_2_O_3_ and the binding energy of the Ga-O bond. As substrate temperature decreases, increasingly more Ga-O bonds are unable to obtain sufficient bonding energy. This results in a smaller grain size, an increased number of grain boundaries, an increased number of grain boundary distortions, and a lower crystalline quality in the film. Compared with sample C, sample D shows slight changes in bandgap and FWHM. This is due to the decomposition of some α-Ga_2_O_3_ grains in the film with the substrate temperature increasing, which results in reduced grain size. Meanwhile, the substrate temperature reaches the critical value corresponding to the formation energy of ε-Ga_2_O_3_, so the diffraction peak of ε-Ga_2_O_3_ appears in the XRD results.

**Figure 4:**
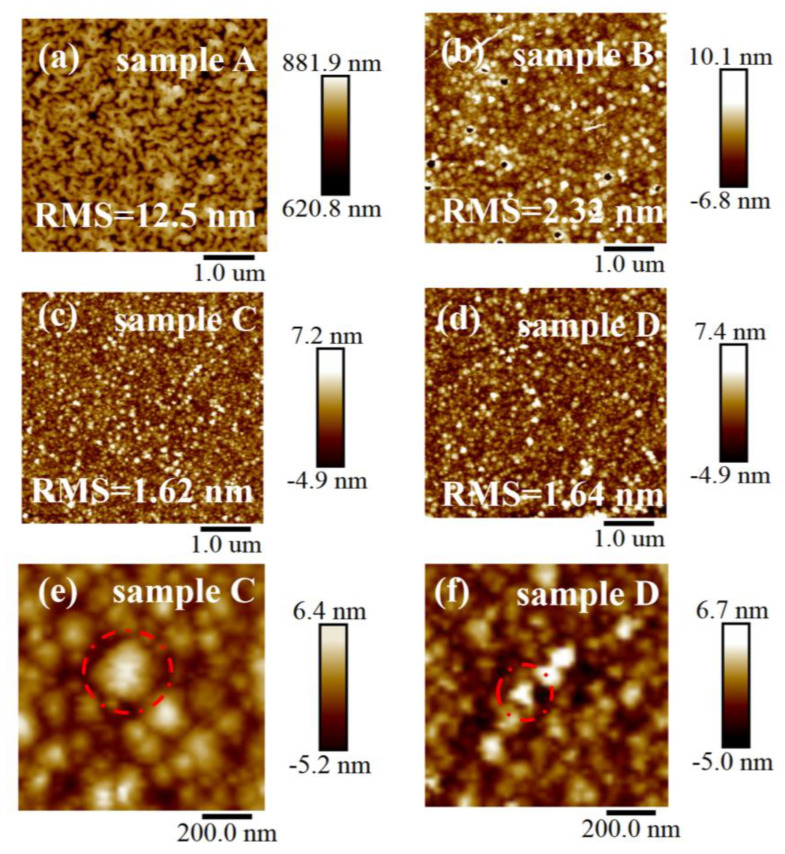
AFM images of the α-Ga_2_O_3_ films grown at different temperatures: (**a**) 400 °C, (**b**) 420 °C, (**c**) 440 °C, (**d**) 460 °C. High-magnification AFM images: (**e**) sample C, (**f**) sample D.

The surface morphologies of samples A, B, C, and D were analyzed using atomic force microscopy (AFM), as shown in Figure 4a–d. As can be seen in Figure 4a, the surface morphology for the sample grown with 400 °C is rough and composed of irregular stripes. It has an RMS surface roughness value of 12.5 nm, which is an order of magnitude more than the value of the other samples. This is due to the fact that the reaction atoms cannot obtain sufficient kinetic energy under low temperature growth. As a consequence, they cannot migrate to the appropriate lattice sites, resulting in the synthesis of large-size grains and subsequently increased roughness. In Figure 4b, the surface morphological composition of the sample changes from irregular strips to grains, and small black spots appear on the surface of the sample. Meanwhile, the root mean square (RMS) roughness decreases from 12.5 nm to 2.32 nm. The AFM images of the samples prepared at 440 °C and 460 °C are shown in Figure 4c,d, and their high-magnification AFM images are shown in Figure 4e,f where the red dashed circle shows the triangular grains. The grain edges on the surface morphology of the sample became clear, and the grains were triangular in shape. However, in Figure 4c, the film surface grain size is more uniform and has lower roughness than other samples.

**Figure 5:**
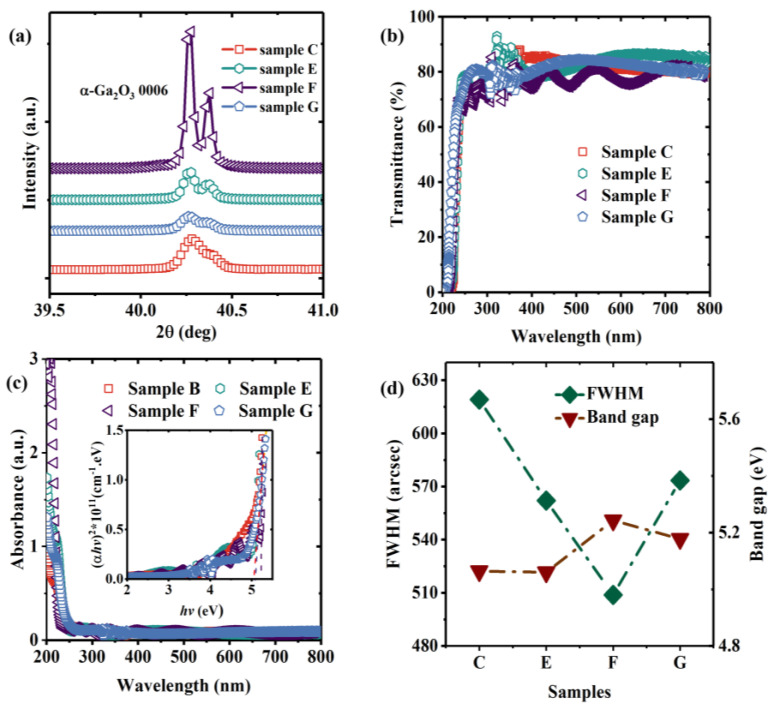
(**a**) XRD θ-2θ diffraction profiles for α-Ga_2_O_3_ films grown under different carrier gas flow rates and types; (**b**) optical transmittance, (**c**) optical absorption spectra, (**d**) bandgaps and FWHMs of α-Ga_2_O_3_ films.

Figure 5a shows the XRD 2θ diffraction profiles for scanning of samples C, E, F, and G which were prepared at the same growth temperature with different carrier gas flow rates and types. For samples C, E, and F, the intensity of the diffraction peaks increases with the carrier gas flow rate rising. Compared with sample F, sample G is prepared under nitrogen, and the intensity of the diffraction peak is significantly lower. Unlike the more obvious strain introduced by the change in temperature, the change in carrier gas flow rate can not cause any significant shift in the positions of the diffraction peaks of samples C, E, F, and G, which indicates that the change of carrier gas flow rate and oxygen content will not cause more obvious plane strain difference in the (0001) direction. 

Figure 5b presents the analysis of the optical transmittance spectra (200–800 nm) of samples C, E, F, and G and shows that the absorption edge of the film redshifts with increasing carrier gas flow rate and oxygen content. For sample F, the absorption edge appears at about 235 nm, and an obvious ripple can be observed which is caused by the interference of reflections from the film surfaces and the interfaces between the epilayer and the substrate. The analysis of optical absorption spectra of the four sample groups is shown in Figure 5c, and the optical bandgaps can be obtained by fitting according to Formula (3) as shown in Figure 5d, which also indicates the FWHM values of the α-Ga_2_O_3_ (0006) plane diffraction peaks for samples C, E, F, and G. As the carrier gas flow rate increases, the optical bandgap of the samples rises to a peak of 5.24 eV (sample F) which is close to the α-Ga_2_O_3_ bandgap figure of 5.30 eV reported in [33]. When the carrier gas changes from air to nitrogen, the bandgap also decreases. However, the value of B_F-G_ (the bandgap of sample F minus the bandgap of sample G) is considerably smaller than the value of B_F-E_, which indicates that the carrier gas flow rate has a greater effect than the carrier gas oxygen content on the bandgap. The carrier gas flow rate affects the amount of reaction source entering the reactor during growth. When the carrier gas flow rate is slow, there are not enough reaction sources available to form crystals in the same crystallization time, which will increase the number of dangling bonds in the film. When the carrier gas flow rate increases, the time taken for the reaction source to enter the reactor approaches the value of the crystallization time which will reduce the number of dangling bonds in the film and improves the crystallization quality. In addition, when the carrier gas flow rate is higher than 5000 sccm, the reaction sources accumulate in the reactor and form amorphous substances on the film surface due to the limitation of exit velocity. 

**Figure 6:**
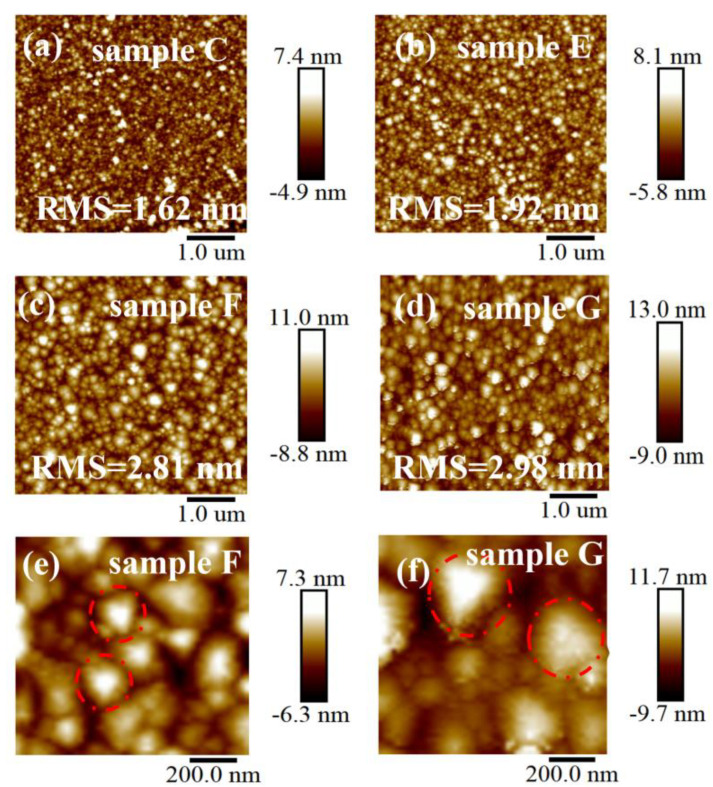
AFM images of the α-Ga_2_O_3_ films grown at different carrier gas flow rates: (**a**) 3000 sccm, (**b**) 4000 sccm, (**c**) 5000 sccm, and (**d**) 5000sccm, N_2_ as carrier gas. High-magnification AFM images: (**e**) sample F, (**f**) sample G.

Figure 6a–d presents the results for AFM analysis of the surface morphology of samples C, E, F, and G, respectively. Figure 6e and 6f show high-magnification AFM images of sample F and G where the red dashed circle shows the triangular grains. When the carrier gas flow rate increases, the grain size on the film surface grows larger, and triangular edges can be observed on most grains. Meanwhile, when grain size increases, the RMS value of the film surface increases from 1.62 to 2.81 nm. This is because the increase of carrier gas flow rate causes transmission of more gallium sources to the substrate surface such that there is a more suitable ratio of the oxygen source and gallium source in the reactor. The quality of α-Ga_2_O_3_ films grown under such conditions is higher, and the increase in crystallinity also leads to an increase in grain size. Therefore, the film surface roughness increases with an increase of the carrier gas flow rate. Figure 6c,d analyzes the surface morphology of samples F and G. Clear-edge grains that can be seen on the surface of the film grown in air, and grains of most sizes are triangular. Compared with the films deposited under air, the grain on the surface of sample G is more inhomogeneous which means that the heterogeneity increased in the surface grain sizes of the films deposited under N_2_. 

## 4. Conclusions

Using simple and cost-effective face-to-face heating mist-CVD equipment, we grew α-Ga_2_O_3_ film on c-sapphire substrates. The surface morphology, optical properties, and structural characteristics of the α-Ga_2_O_3_ films were investigated. Under optimized growth conditions, the FWHM of the α-Ga_2_O_3_ 0006 plane diffraction peak was 508 arcsec, the optical bandgap measured by the optical absorption spectrum reached 5.24 eV, and the RMS surface topography value reached 2.98 nm. Although the quality of the films prepared by our method was slightly inferior compared with those produced by the method described in [34], the cost of production of our films was substantially lower. This may be beneficial to future research and the promotion of α-Ga_2_O_3_. In future work, we will focus on improving our film quality.

## Figures and Tables

**Table 1:** Film growth conditions.

Sample	Temperature		Carrier Gas	
	Down (°C)	Up (°C)	Flow Rate (sccm)	Type
A	400	420	3000	Air
B	420	440	3000	Air
C	440	460	3000	Air
D	460	480	3000	Air
E	440	460	4000	Air
F	440	460	5000	Air
G	440	460	5000	N_2_

## Data Availability

The data presented in this study are available on request from the corresponding author.
